# Supplementary material for: HPV Infection Significantly Accelerates Glycogen Metabolism in Cervical Cells with Large Nuclei: Raman Microscopic Study with Subcellular Resolution
Source: Int J Mol Sci. 2020 Apr 11;21(8):2667. doi: 10.3390/ijms21082667 (PMC7215571; doi:10.3390/ijms21082667)
Supplement: Supplementary file 1 [file ijms-21-02667-s001.pdf]

Supplementary Materials

HPV infection significantly accelerates glycogen metabolism in cervical cells with large nuclei: Raman microscopic study with subcellular resolution

Katarzyna Sitarz [1,2], Krzysztof Czamara [3], Joanna Bialecka [4], Malgorzata Klimek [5], Barbara Zawilinska [1], Slawa Szostek [1*] and Agnieszka Kaczor [2,3*]

[1] Jagiellonian University Medical College, Faculty of Medicine, Chair of Microbiology, Department of Virology, 18 Czysta Street, 31-121 Krakow, Poland.
[2] Jagiellonian University, Faculty of Chemistry, 2 Gronostajowa Street, 30-387 Krakow, Poland.
[3] Jagiellonian University, Jagiellonian Centre for Experimental Therapeutics (JCET), 14 Bobrzynskiego Street, 30-348 Krakow, Poland.
[4] Centre of Microbiological Research and Autovaccines, 17 Slawkowska Street, 31-016 Krakow, Poland.
[5] Maria Sklodowska-Curie Institute – Oncology Center, Clinic of Radiotherapy, 11 Garncarska Street, 31-115 Krakow, Poland* Correspondence: Agnieszka Kaczor (agnieszka.kaczor@uj.edu.pl) and Slawa Szostek (slawa.szostek@uj.edu.pl)

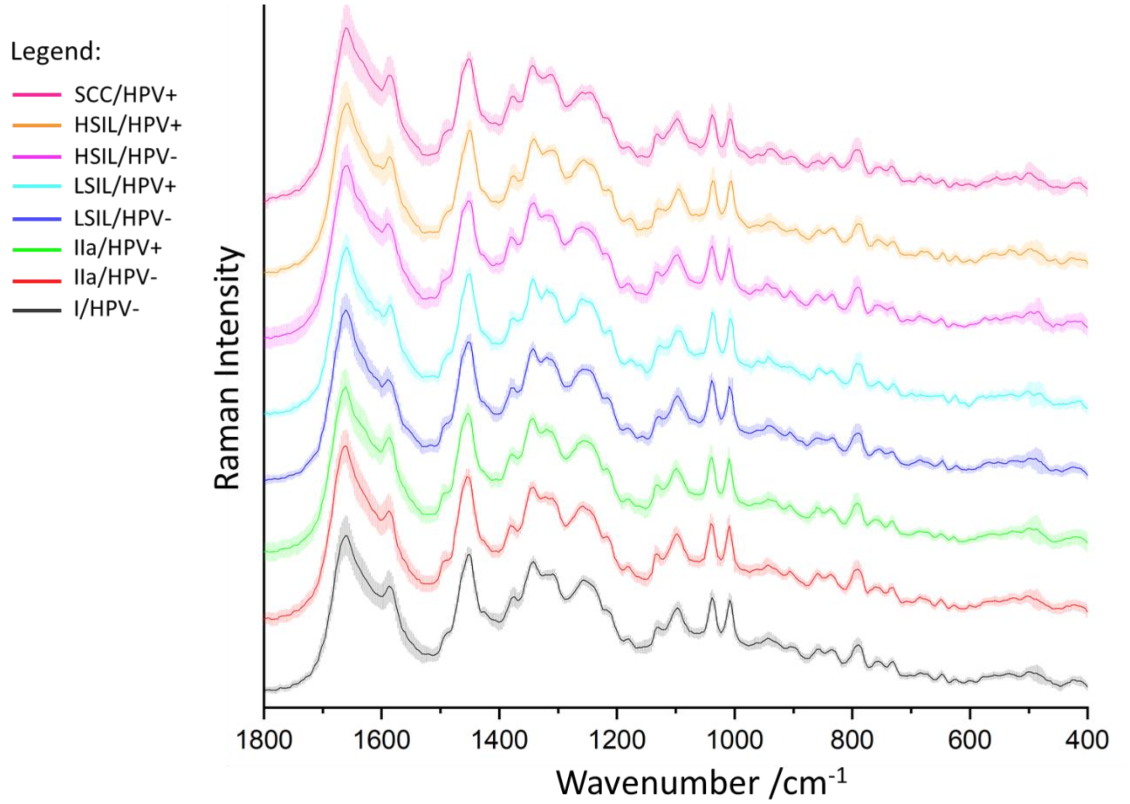

Figure S1. Raman average spectra of nuclei in cervical cells in studied groups of females. Presented Raman spectra were averaged over all nuclei in the studied group and the nuclei were separated based on Cluster Analysis (K-means, Manhattan distance).

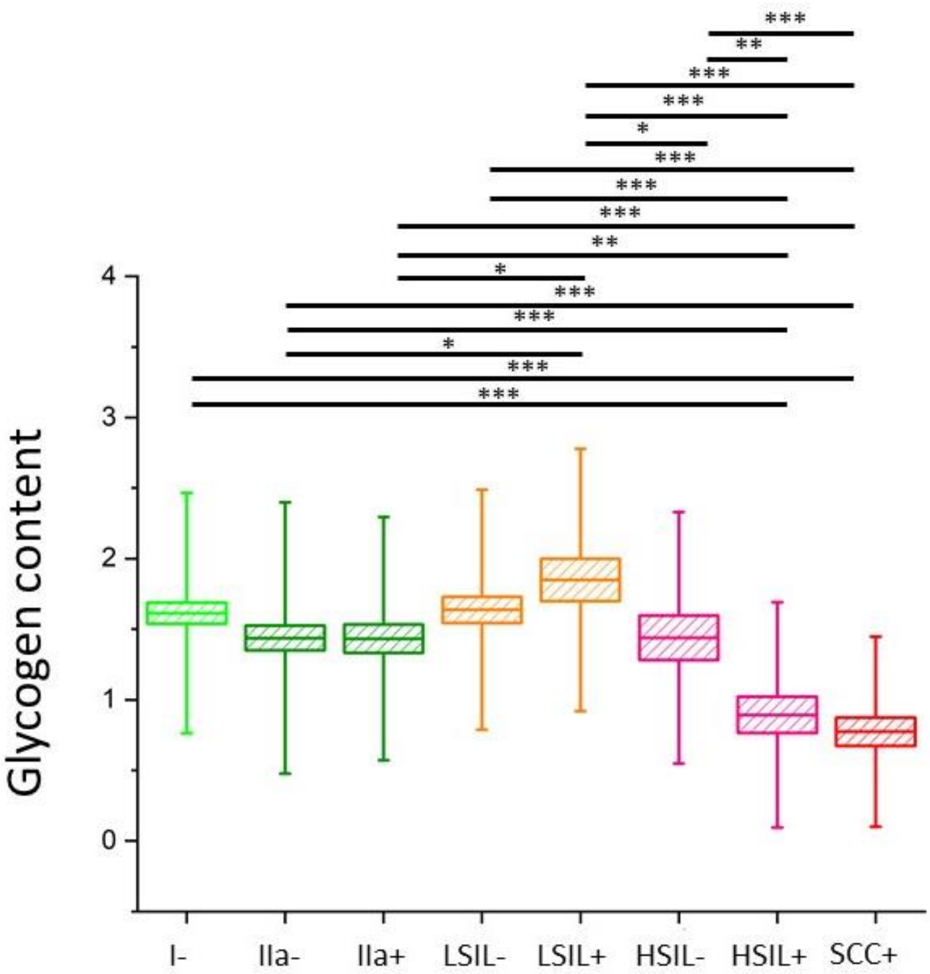

Figure S2. Glycogen content in the cytoplasm of cervical epithelial cells. The comparison of the glycogen content in the cytoplasm of cervical epithelial cells in studied groups: I/HPV- (bright green), IIa/HPV- (dark green), IIa/HPV + (dark green), LSIL/HPV- (orange), LSIL/HPV+ (orange), HSIL/HPV- (pink), HSIL/HPV+ (pink), SCC/HPV+ (red) obtained by calculations of the integral intensity of the glycogen marker band at 486 cm-1 in studied cells (in total 560). Mean values ± SEM are given as box plots: mean (horizontal line), SEM (box), minimal and maximal values (whiskers). * p < 0.05 ** p < 0.01 *** p < 0.001.

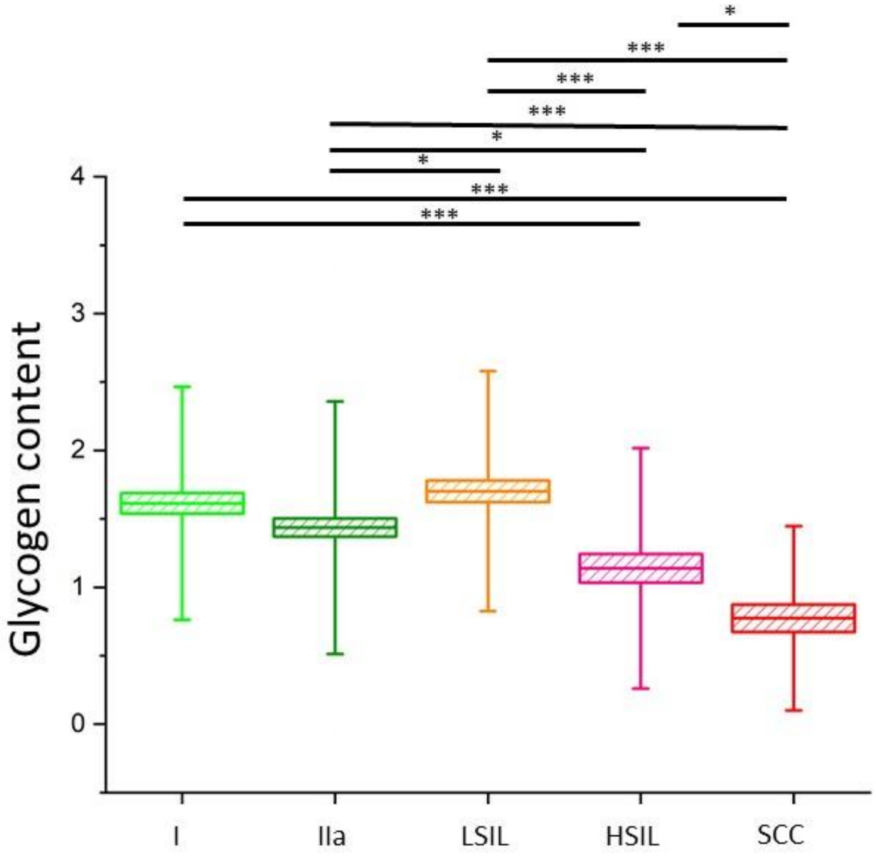

Figure S3. Glycogen content in the cytoplasm of cervical epithelial cells without HPV-infection based differentiation. The comparison of the glycogen content in the cytoplasm of cervical epithelial cells in studied groups: I (bright green), IIa (dark green), LSIL (orange), HSIL (pink), SCC (red) obtained by calculations of the integral intensity of the glycogen marker band at 486 cm-1 in studied cells (in total 560). Mean values ± SEM are given as box plots: mean (horizontal line), SEM (box), minimal and maximal values (whiskers). * $p < 0.05$ *** $p < 0.001$.

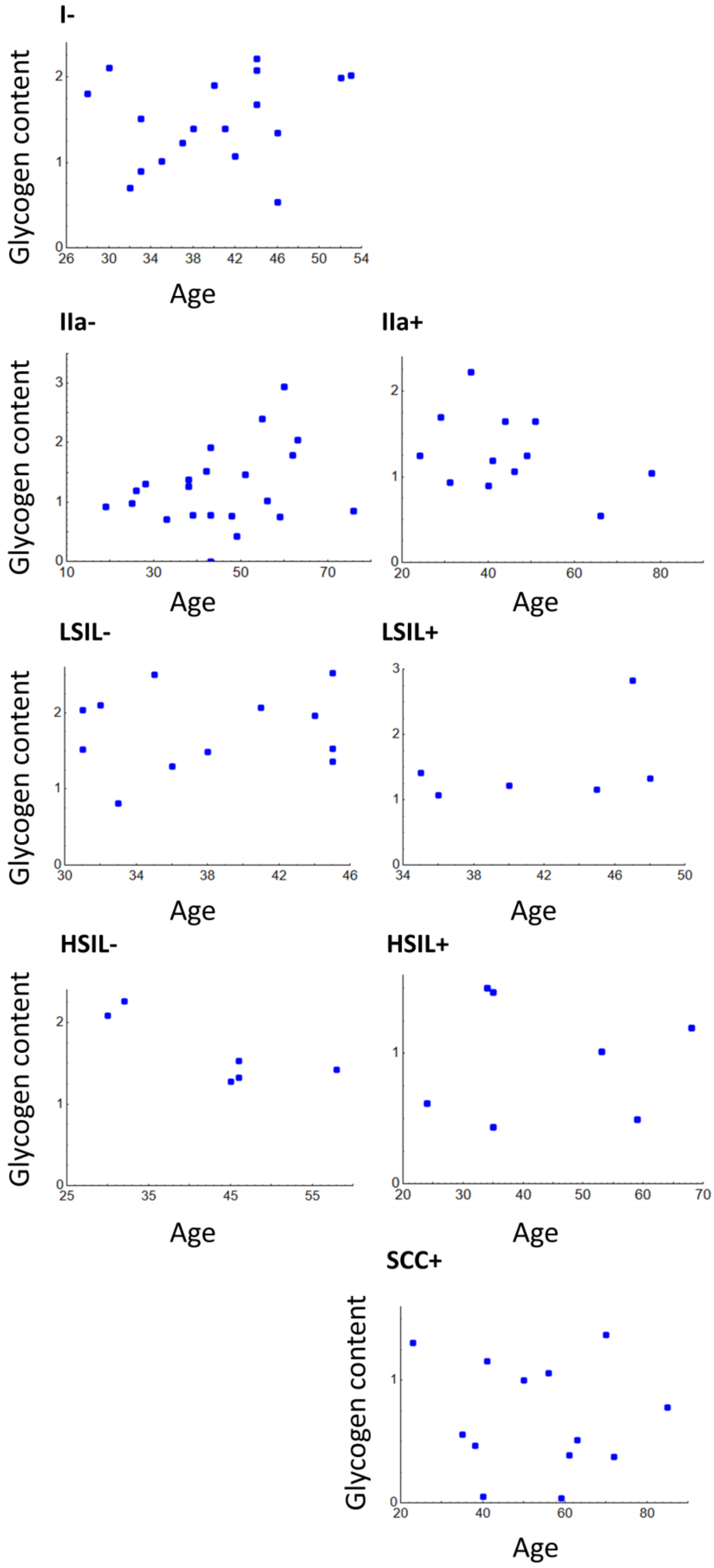

Figure S4. Glycogen content in the cytoplasm of cervical epithelial cells as a function of age. Analysis of the correlation between the average amount of glycogen (obtained by calculations of the integral intensity of the glycogen marker band at 941 cm^{-1}) in the cytoplasm of cells and the age of females in individual groups. Each blue circle represents average data for one patient. Graphs present individual groups of patients: I/HPV- (A), IIa/HPV- (B), IIa/HPV+ (C), LSIL/HPV- (D), LSIL/HPV+ (E), HSIL/HPV- (F), HSIL/HPV+ (G), SCC/HPV+ (H).

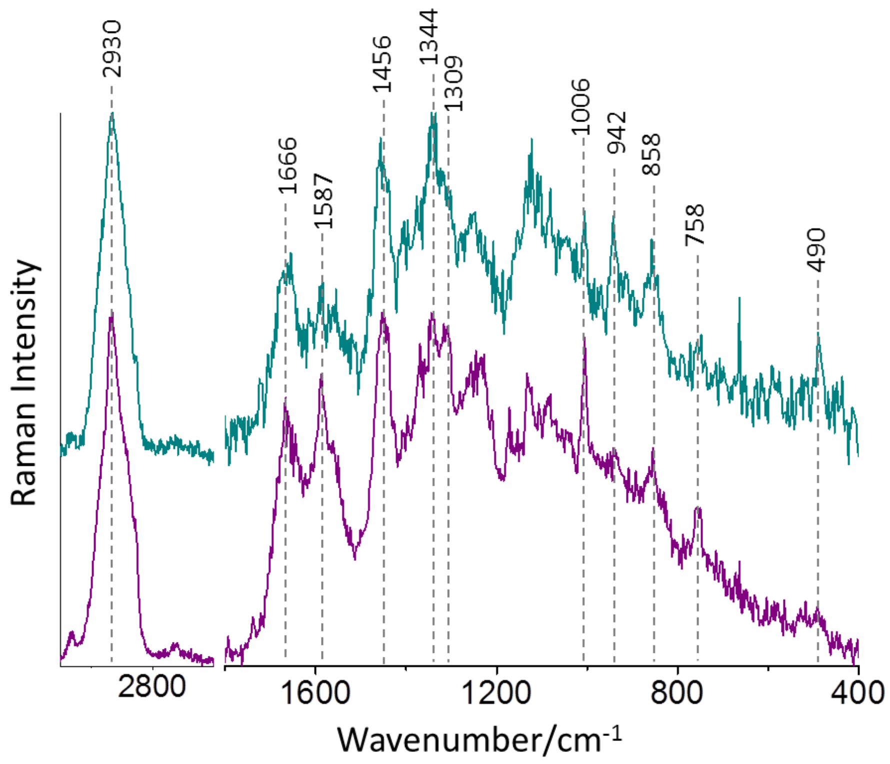

Figure S5. Single Raman spectra of dry pellets of cervical epithelial cells of two patients differing in the glycogen level obtained using fiber optic Raman spectroscopy. Spectra were acquired with a novel portable Raman fiber optic probe (WITec, Germany) using the 532 nm laser excitation. Each spectrum was accumulated with the 4 s integration time and 10 accumulations.

Table S1. The number of patients in studied groups.

Analyzed group	HPV status	Number of patients
I	HPV-<br>HPV+	18<br>0
IIa	HPV-<br>HPV+	22<br>12
IIID-1=LSIL[a]	HPV-<br>HPV+	12<br>6
IIID-2=HSIL[b]	HPV-<br>HPV+	6<br>7
SCC[c]	HPV-<br>HPV+	0<br>13

[a] low-grade squamous intraepithelial lesion

[b] high-grade squamous intraepithelial lesion

[c] histopathologically confirmed squamous cell carcinoma

Table S2. Glycogen content in studied epithelial cervical cells. Glycogen content calculated based on the relative intensity of the Raman band at 486 cm^{-1}.

Classification	Number of cells	Relative glycogen content	Statistical significance
Cells with small-diameter nuclei			
**I/HPV-**	83	Mean: 1.75<br>SD: 0.84<br>SE: 0.09	LSIL/HPV+***<br>HSIL/HPV+*<br>SCC/HPV+***
**IIa/HPV-**	65	Mean: 1.61<br>SD: 1.00<br>SE: 0.12	LSIL/HPV+***<br>SCC/HPV+***
**IIa/HPV+**	55	Mean: 1.69<br>SD: 0.81<br>SE: 0.11	LSIL/HPV+***<br>HSIL/HPV+*<br>SCC/HPV+***
**LSIL/HPV-**	68	Mean: 1.65<br>SD: 0.85<br>SE: 0.10	LSIL/HPV+***<br>HSIL/HPV+*<br>SCC/HPV+***
**LSIL/HPV+**	24	Mean: 2.38<br>SD: 0.69<br>SE: 0.14	I/HPV-***<br>IIa/HPV-***<br>IIa/HPV+***<br>LSIL/HPV-***<br>HSIL/HPV-**<br>HSIL/HPV+***<br>SCC/HPV+***

**HSIL/HPV-**	18	Mean: 1.70<br>SD: 0.73<br>SE: 0.17	LSIL/HPV+**<br>HSIL/HPV+*<br>SCC/HPV+***
**HSIL/HPV+**	17	Mean: 1.16<br>SD: 0.92<br>SE: 0.22	I/HPV-*<br>IIa/HPV+*<br>LSIL/HPV-*<br>LSIL/HPV+***<br>HSIL/HPV-*<br>SCC/HPV+*
**SCC/HPV+**	21	Mean: 0.56<br>SD: 0.55<br>SE: 0.12	I/HPV-***<br>IIa/HPV-***<br>IIa/HPV+***<br>LSIL/HPV-***<br>LSIL/HPV+***<br>HSIL/HPV-***<br>HSIL/HPV+*
**Cells with large-diameter nuclei**			
**I/HPV-**	43	Mean: 1.35<br>SD: 0.82<br>SE: 0.12	IIa/HPV+**<br>HSIL/HPV+***<br>SCC/HPV+ *
**IIa/HPV-**	56	Mean: 1.23<br>SD: 0.88<br>SE: 0.12	IIa/HPV+*<br>HSIL/HPV+ **
**IIa/HPV+**	19	Mean: 0.68<br>SD: 0.49<br>SE: 0.11	I/HPV-**<br>IIa/HPV-*<br>LSIL/HPV-**
**LSIL/HPV-**	17	Mean: 1.57<br>SD: 0.87<br>SE: 0.21	IIa/HPV+ **<br>LSIL/HPV+*<br>HSIL/HPV+**<br>SCC/HPV+*
**LSIL/HPV+**	14	Mean: 0.95<br>SD: 0.48<br>SE: 0.13	LSIL/HPV-*<br>HSIL/HPV+*
**HSIL/HPV-**	14	Mean: 1.10<br>SD: 0.99<br>SE: 0.26	-
**HSIL/HPV+**	22	Mean: 0.69<br>SD: 0.63<br>SE: 0.13	I/HPV-***<br>IIa/HPV-**<br>LSIL/HPV-**<br>LSIL/HPV+*
**SCC/HPV+**	24	Mean: 0.97<br>SD: 0.72<br>SE: 0.15	I/HPV-*<br>LSIL/HPV-*

*p<0.05**p<0.01***p<0.001

Table S3. Glycogen content in studied epithelial cervical cells. Glycogen content calculated based on the percentage of cells stained with the PAS method.

Classification	Number of samples	Relative glycogen content	Statistical significance
**I**	10	Mean: 70.1<br>SD: 8.7<br>SE: 2.9	HSIL**<br>SCC*
**IIa**	13	Mean: 64.2<br>SD: 8.6<br>SE: 2.4	HSIL**<br>SCC*
**LSIL**	13	Mean: 62.2<br>SD: 15.1<br>SE: 4.2	HSIL**<br>SCC*
**HSIL**	5	Mean: 24.6<br>SD: 22.8<br>SE: 10.2	I**<br>IIa**<br>LSIL**
**SCC**	3	Mean: 38.3<br>SD: 11.7<br>SE: 6.2	I*<br>IIa*<br>LSIL*

*p<0.05**p<0.01
